# Pesticides’ Cornea Permeability—How Serious Is This Problem?

**DOI:** 10.3390/pharmaceutics17020156

**Published:** 2025-01-24

**Authors:** Anna W. Sobańska, Andrzej M. Sobański, Karolina Wanat

**Affiliations:** 1 Department of Analytical Chemistry, Medical University of Lodz, Muszynskiego 1, 90-151 Lodz, Poland; karolina.wanat@umed.lodz.pl; 2 Faculty of Chemistry, University of Lodz, Tamka 12, 91-403 Lodz, Poland; andrzej.sobanski@edu.uni.lodz.pl

**Keywords:** cornea permeability, MLR models, ANN models, eye corrosion, pesticides

## Abstract

**Background:** A total of 348 pesticides from different chemical families (carbamates, organochlorines organophosphorus compounds, pyrethroids, triazines and miscellaneous) were investigated in the context of their cornea permeability and potential to cause eye corrosion. **Methods:** Multivariate models of cornea permeability based on compounds whose cornea permeability has been determined experimentally were proposed. The models, applicable to compounds across a relatively broad lipophilicity range (e.g., pesticides with octanol–water partition coefficient log ***P*** up to ca. 8), assume a reverse-parabolic relationship between cornea permeability and lipophilicity, expressed as ***XLOGP3***; other main descriptors present in the models are log ***D*** at pH 7.4 and polar surface area (***PSA***). **Results:** It appears that the trans-corneal transport of all studied pesticides is possible to some degree; however, it is more difficult for the majority of highly lipophilic pesticides from the organochlorine and pyrethroid families. The same set of 348 pesticides was also evaluated for their eye-corrosive potential using novel artificial neural network models involving simple physico-chemical properties of the compounds (lipophilicity, aqueous solubility, polar surface area, H-bond donor and acceptor count and the count of atoms such as N, NH, O, P, S and halogens). **Conclusions:** It was concluded that eye corrosion is an issue, especially among the pesticides from organochlorine and organophosphorus families.

## 1. Introduction

The cornea is the outermost layer of the eye, controlling the entry of light into the eye and shielding the inner parts of the eyeball against germs, dust and other harmful factors [1]. It consists of three main layers: endothelium, stroma and epithelium. One of them—the endothelium—plays no significant role in preventing compounds’ passage across the cornea; it is a monolayer of hexagonal cells that can be crossed either by a paracellular or transcellular route, favored by hydrophilic/ionic and lipophilic compounds, respectively [2]. The other cornea layers are rate-limiting barriers for lipophilic (aqueous stroma) and hydrophilic (lipophilic corneal epithelium) compounds, respectively. Ophthalmic drugs, if they are to be administered the easiest way—as eyedrops or ointments—should be able to cross the cornea; compounds that are not absorbed via the cornea may enter the eye through the conjunctiva, but this is a minor route, available for large, hydrophilic molecules (e.g., peptides or proteins) [3]. If the corneal permeability of typical, small-molecule drugs is too low and they cannot achieve required concentrations in the eye, they may be taken systemically (in relatively high doses to account for the drugs’ metabolism and distribution in other tissues and with all possible side effects) or by intravitreal injections; in the latter case, drugs (used, e.g., for macular degeneration) are injected directly into the eyeball, which is also associated with some potential side effects, especially after repeated applications [4]. Some drugs may be administered by periocular routes to different spots in the area immediately surrounding the eye, which reduces the risks associated with intravitreal injections and systemic administration [5]. Following periocular delivery, compounds move to the sclera and undergo transscleral absorption [4] (a detailed review of the permeability of the sclera, conjunctiva and different layers of the cornea to small groups of compounds is given in ref. [6]).

Factors that influence the passage of compounds across the cornea are physiological, physico-chemical and formulation-related. In drug design, the main focus is on the physico-chemical properties of drug candidates to facilitate trans-corneal absorption. Early observations pointed to the relationship between the chain length and the corneal permeability for n-alkyl p-aminobenzoate esters [7]. As early as the 1970s and 1980s, it was also established that the corneal permeability of drugs depends on their lipophilicity and this relationship is either parabolic, with the maximum permeability at log ***P*** (or log ***D***) between 2 and 3 [8,9], or sigmoidal [10,11]. Later, it was discovered that other physico-chemical properties are also of importance [12,13]. New “Rules of Thumb” for ophthalmic drugs have been proposed recently and the physico-chemical parameters found to facilitate drugs’ topical ophthalmic delivery are the distribution coefficient at physiological pH, topological polar surface area and free energy of octanol–water distribution below certain limits (clog ***D***_pH7.4_ < 4.0; ***TPSA*** < 250 Å^2^; Δ***G***_o/w_ < 20 kJ/mol) and sufficient aqueous solubility (>1 mM) [14].

Due to the asymmetry in the cornea structure, the transport of compounds across the cornea (inwards/outwards) is also asymmetrical, with additional phenomena such as tear drainage reducing compounds’ eye penetration [13,15]; in the case of drugs, not more than 5% of the applied dose reaches the intraocular tissues [3]. Compounds that reach the vitreous cavity are eliminated via blood–ocular barriers (blood–aqueous and blood–retinal barriers) [16,17].

Drug candidates can be examined for their corneal permeability by animal experiments in vivo [18] and ex vivo animal models [11,19,20,21,22,23] or, to avoid ethical controversies, the ability of compounds to cross the cornea can be studied in vitro—by cell/tissue-based assays [24,25,26,27,28] or by non-cell-based methods, e.g., the parallel artificial membrane permeability assay (PAMPA) [29] or liquid chromatographic models [30,31]—or in silico [1,11,32,33,34,35,36,37,38,39,40].

At present, a lot of attention is being given to the ocular toxicity of environmental contaminants contributing to ophthalmic conditions such as eye irritation, dry eye syndrome, etc. Anthropogenic chemicals from both civilian and military sources (e.g., contaminants from burn pits and train derailment; riot control agents; illicit drugs; herbicides, etc.) were reviewed in relation to the eye, and their negative influence upon different eye elements (cornea, retina or optic nerve) after topical and systemic exposures was discussed [41,42,43,44].

The objective of this study is to assess the ability of an important class of environmental pollutants—pesticides—to cross the cornea and to evaluate compounds from this group as possible causes of eye corrosion using novel in silico methods based on experimental data.

## 2. Materials and Methods

### 2.1. Reference Compounds

Compounds **1** to **90**, along with their experimental corneal permeability coefficients, log ***K***_corn_, were taken from [1,6,34]. Compounds **91** to **2389**, with experimental data related to eye corrosion used to train, test and validate models of eye-corrosive activity, were taken from [45].

### 2.2. Molecular Descriptors

Electrotopological state indices [46,47], polar surface area (***PSA***), counts of particular atoms (***NH***, ***O***, ***N***, ***DONORS***, ***ACCEPTORS***, ***HALOG***, ***P*** and ***S***), aqueous solubility (***ALogPS_logS***) and octanol–water partition coefficients (***ALogPS_logP***) were calculated online using the OChem platform (https://ochem.eu, accessed on 30 November 2024). Octanol–water distribution coefficient at pH = 7.4 (log ***D***_ACD_) were calculated using ACDLabs software (Version 7.0). Octanol–water partition coefficients were also calculated using ACDLabs software (log ***P***_ACD_) and SwissADME software (http://www.swissadme.ch/, accessed on 30 November 2024) [48] (***XLOGP3***, ***iLOGP***, ***MLOGP***, ***WLOGP***, ***Silicos-IT Log P*** and ***Consensus Log P***). Molecular properties of the studied compounds, including molecular weight (***MW***), volume (***Vol***), H-bond donor count (***nHD***), H-bond acceptor count (***nHA***), formal charge (***fChar***), rigid bond count (***nRig***), rotatable bond count (***nRot***), flexibility = ***nRot***/***nRig*** (***Flex***), stereocenter count (***nStereo***), maximum ring size (***MaxRing***), number of heteroatoms, i.e., non-carbon atoms including hydrogens (***nHet***)**, ** fraction of sp^3^ carbons (***F_Csp3_***) and topological surface area (***TPSA***), were computed using ADMETlab3.0 software (https://admetlab3.scbdd.com/, accessed on 30 November 2024) [49]. Predicted absorption properties, including parallel artificial membrane permeability assay (***PAMPA***), Madin−Darby canine kidney cells’ logarithmic permeability coefficient (***MDCK***) and the human colon adenocarcinoma cell lines’ permeability (***Caco-2***) were also calculated using ADMETlab3.0 software.

### 2.3. Multiple Linear Regression (MLR) Models of Corneal Permeability

Compounds **1** to **60** were used as a training set and **61** to **90** as a test set. MLR models were generated using XLSTAT (Lumivero (2025) XLSTAT statistical and data analysis solution, https://www.xlstat.com/en). Descriptors with zero variability were removed manually and the suitable descriptors for MLR models were selected using stepwise regression, in forward mode. Equations (8), (9) and (14)–(16) were subjected to internal and external validation using the following metrics: determination coefficient of the training set (R^2^); root mean square error of leave-many-out (LMO) cross-validation (5-fold; RMSECV); root mean square error of external prediction (RMSEP); and cross-validated R^2^ (Q^2^) [50,51].

### 2.4. Artificial Neural Network (ANN) Classification of Pesticides with High (1) and Low (0) Risk of Eye Corrosion

*Compounds* **91** to **2389** were randomly divided into three sets for training, testing and validation (70, 15 and 15% of the whole set, respectively), as indicated in the Supplementary Materials. Multilayer perceptron (MLP) artificial neural networks (ANNs) were generated using Statistica v. 13.3 (classification mode, Automated Network Search (ANS) module, 100 networks to train, 5 networks to retain). The possible neuron activation functions were as follows: identity, logistic, hyperbolic tangent and exponential. The BFGS (Broyden–Fletcher–Goldfarb–Shanno) algorithm was used to train the network together with the sum of squares (SOS) or entropy error function. After the networks were generated, all independent variables were examined using their Global Sensitivity Analysis scores to check if they are of sufficient importance to remain in the model (GSA scores rate the contribution of particular variables to the dependent variable being modeled; variables with GSA scores below 1 are redundant, as the model performs equally well or better without them).

## 3. Results and Discussion

### 3.1. Cornea Permeability

It was reported in 1979 that the corneal permeability of PABA esters depends on the number of carbon atoms in the n-alkyl chain (and this relationship is reverse parabolic, with the maximum cornea permeability obtained for the propyl ester) [7]. Following this observation, the relationships between cornea permeability and different physico-chemical properties of compounds have been studied extensively. The cornea permeability of compounds has been predicted using the QSAR methodology by several authors thus far, with the most distinguished models listed below in Equation (1) [1], Equations (2) and (3) [37], Equations (4) and (5) [31], Equation (6) [38] and Equation (7) [40]:

log ***K***_corn_ = −0.005 ***MW*** − 0.166 ***HBD*** − 0.081 (±0.075) ***nHA*** + 0.164 log ***P*** + 0.209 log ***D*** − 5.994 ***ıH(max)*** − 3.740 (n = 47, R**^2^** = 0.77) (1)

log ***K***_corn_ = 0.390 log ***P*** − 0.00327 ***MW*** − 4.77 (n = 105, R^2^ = 0.51) (2)

log ***K***_corn_ = 0.230 log ***P*** − 0.0679 ***n_H_*** − 0.168 ***κ3*** − 4.35 (n = 95, R^2^ = 0.52) (3)

log ***K***_corn_ = −4.4 + 0.83 log ***k***_BMC_ − 0.0044 ***MW*** (n = 38, R^2^ = 0.80) (4)

log ***K***_corn_ = −4.0 + 0.25 log ***D*** − 0.0040 ***MW*** (n = 38, R^2^ = 0.57) (5)

log ***K***_corn_ = −5.566 ***Q***_H_ 2 + 3.027 ***Q***_H_ − 0.155 ***Q***_O,N_ − 9.413 × 10^−4^ ***V*** − 4.278 (n = 30, R^2^ = 0.85) (6)

log ***K***_corn_ = −3.885 − 0.183 ***nHB***_tot_ + 0.277 log ***D*** (n = 58, R^2^ = 0.78) (7)

**Figure 1:**
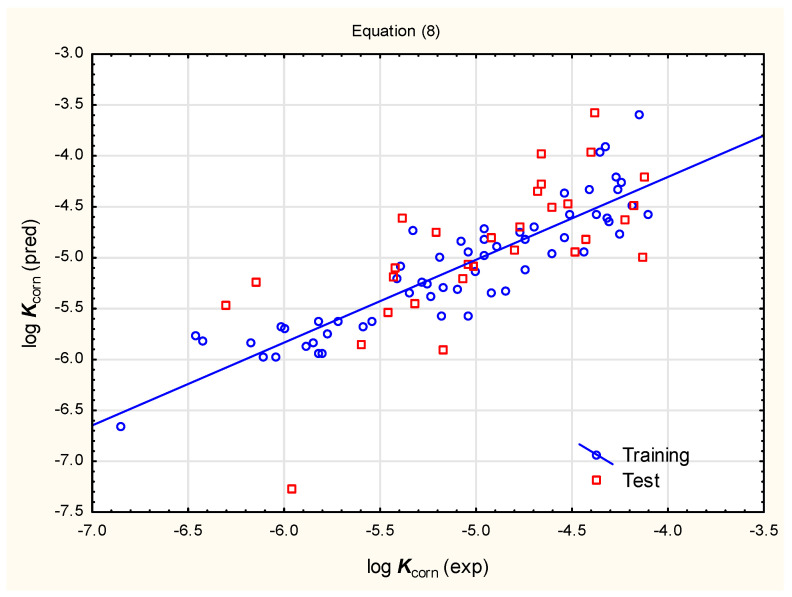
Predicted vs. observed log ***K***_corn_ for compounds **1** to **90**—Equation (8).

**Figure 2:**
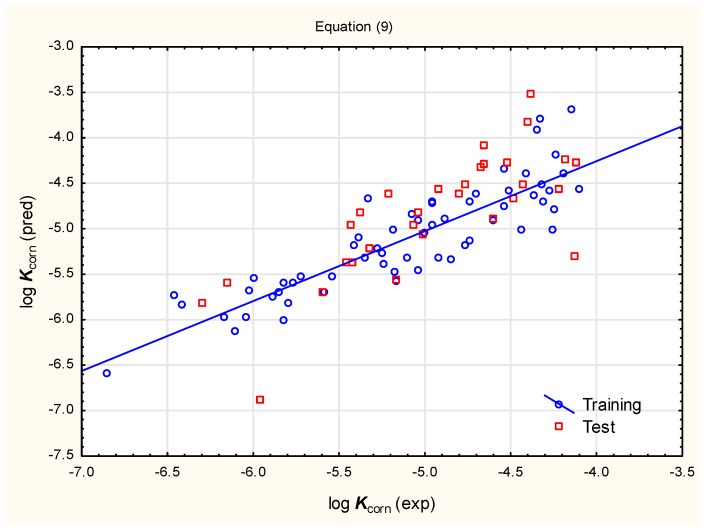
Predicted vs. observed log ***K***_corn_ for compounds **1** to **90**—Equation (9).

Equations (1)–(7) are related to skin permeability models developed by Potts and Guy [52,53], who stressed the positive, linear correlation between compounds’ lipophilicity and skin permeability and who also reported the negative influence of molecules’ size (volume or weight) and the ability to form H-bonds (H-bond donor and acceptor count, and total H-bond count) on their transdermal absorption. Unfortunately, Equations (1)–(7), however valuable, also have certain disadvantages: (i) they have moderate statistics and (ii) the majority of them were developed based on small groups of structurally related compounds (mainly small-molecule drugs), whose physico-chemical properties are within narrow ranges [14], which, as it may be expected, also makes their application domains rather narrow. With these considerations in mind and with the focus on pesticides from several very different chemical families, we have decided to develop new models that are more suitable for compounds with diverse physico-chemical properties. Our attention turned at first to refining existing models based on the linear relationships between log ***K***_corn_ and log ***P***/log ***D***; two multivariate linear (MLR) equations, Equation (8) and a simplified Equation (9), generated in the process are given below (Figure 1 and Figure 2):

log ***K***_corn_ = −4.198 (±0.184) + 0.159 (±0.047) log ***D***_ACD_ − 0.0164 (±0.0026) ***PSA*** + 0.206 (±0.048) ***XLOGP3*** − 0.0397 (±0.0139) ***SaaCH*** + 0.333 (±0.113) ***Se1C1C3a*** + 0.0522 (±0.0256) ***SsNH2*** (n = 60, R^2^ = 0.813, R^2^_adj._ = 0.792, F = 38.49, *p* < 0.01, RMSECV = 0.357, RMSEP = 0.511, Q^2^ = 0.765) (8)

log ***K***_corn_ = −4.366 (±0.169) + 0.209 (±0.045) log ***D***_ACD_ − 0.0125 (±0.0017) ***PSA*** + 0.179 (±0.052) ***XLOGP3*** − 0.0319 (±0.0150) ***SaaCH*** (n = 60, R^2^ = 0.769, R^2^_adj._ = 0.752, F = 45.84, *p* < 0.01, RMSECV = 0.394, RMSEP = 0.452, Q^2^ = 0.722) (9)

Despite their relatively good statistical parameters (including the results of external validation), Equations (8) and (9) were found to be unsatisfactory in the context of their applicability to studied pesticides. The results of log ***K***_corn_ calculations based on Equations (8) and (9) returned suspiciously high results (pointing to very good trans-corneal absorption) for more lipophilic pesticides, especially from the organochlorine and pyrethroid families.

At this point, the decision was made to improve the models by using polynomial expressions. This approach was inspired by some already mentioned early reports on the on-linear relationships between cornea permeability and the lipophilicity-related parameters of compounds: (i) the chain length for n-alkyl p-aminobenzoate esters [7] and (ii) log ***D*** or log ***P*** [8,9,39]. Schoenwald and Huang experimented with quadratic functions of lipophilicity, also incorporating the logarithm of molecular weight (log ***MW***)—the latter was reversely correlated with permeability. They also studied the effects of ionization in the context of trans-corneal transport and proposed equations involving the degree of ionization (log ***DI***) or the distribution coefficient (log ***D***) instead of the partition coefficient (log ***P***) (Equations (10) and (11)), discovering that for a small group of structurally similar compounds, the effect of log ***MW*** is negligible and the quadratic function of log ***D*** accounts for ca. 95% of the total log ***K***_corn_ variability [9]. However, later studies revealed that log ***D*** used on its own is not sufficient as a cornea permeability predictor for larger sets of more structurally diverse drugs [39].

log ***K***_corn_ = 1.01 log ***P*** − 0.115 (log ***P***)^2^ − 5.64 log ***MW*** − 10.4 log ***DI*** + 7.27 (n = 12, R^2^ = 0.860) (10)

log ***K***_corn_ = 0.646 log ***D*** − 0.101 (log ***D***)^2^ − 4.891 log ***MW*** + 6.907 (n = 12, R^2^ = 0.851) (11)

log ***K***_corn_ = −0.071 (log ***D***)^2^ + 0.465 log ***D*** − 5.265 (n = 23, R^2^ = 0.621) (12)

log ***K***_corn_ = −0.084(log ***D***)^2^ + 0.464 log ***D*** − 5.170 (n = 22, R^2^ = 0.663) (13)

Equations (10)–(13) explain the existence of a plateau for log ***K***_corn_ = f (lipophilicity) dependencies observed at log ***P***/log ***D*** = ca. 3, but they have not been tested on compounds with log ***D*** > 4; in fact, the majority of compounds used to develop known models of cornea permeability are small, drug-like molecules, usually meeting the requirements formulated by Karami [14]. However, non-linear (e.g., reverse-parabolic or Gaussian-shaped) relationships between the compounds’ lipophilicity and absorption/bioavailability have been proposed, e.g., for skin permeability, PAMPA permeability [54] or half-maximum inhibitory concentrations (IC50s) [55] of different groups of drugs. The non-linearity of relationships between the studied property and the lipophilicity is especially pronounced for the bioconcentration factor in aquatic organisms (***BCF***). The physico-chemical properties of environmental pollutants are much more diverse than those of drugs, and experimental ***BCF*** values are available for a significant number of compounds over a very broad lipophilicity range [56]. It was found that log ***BCF*** is relatively stable for highly hydrophilic compounds (log ***P*** < 1), increases proportionally to lipophilicity for log ***P*** between 1 and ca. 7 and is reversely proportional to lipophilicity for log ***P*** between 7 and 10, with some negative influence found for molecular size [57,58]; for very highly lipophilic compounds (log ***P*** > 10), the log ***BCF***–lipophilicity relationship is likely to reach another plateau, but experimental ***BCF*** data to support this view are limited.

**Figure 3:**
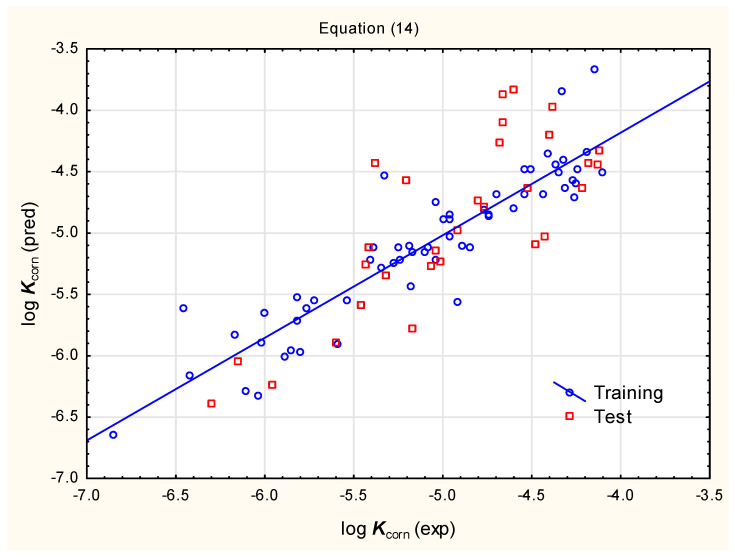
Predicted vs. observed log ***K***_corn_ for compounds **1** to **90**—Equation (14).

**Figure 4:**
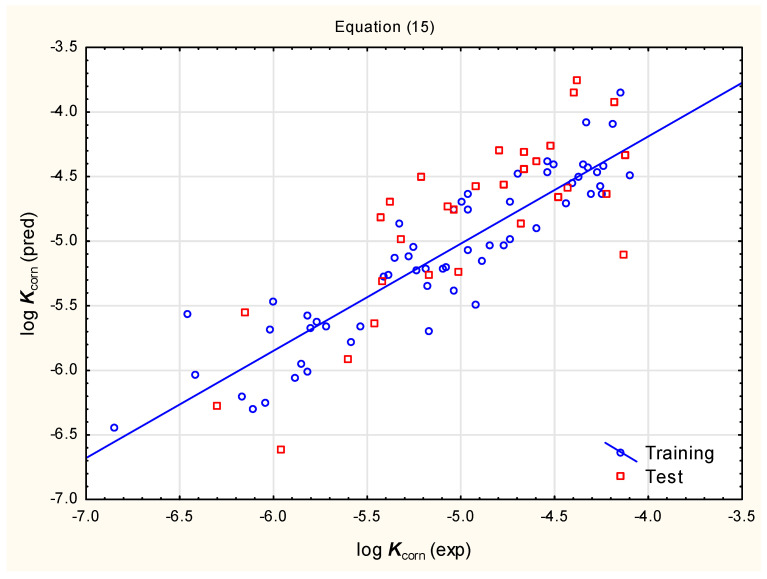
Predicted vs. observed log ***K***_corn_ for compounds **1** to **90**—Equation (15)**.**

As many pesticides investigated in this study are relatively lipophilic (with log ***P*** up to ca. 8), we suspected that (by analogy to other absorption/transport processes mentioned above) very high ***K***_corn_ values obtained for them using Equations (8) and (9) may be artifacts. We sought to account for the possible reduction in cornea permeability for highly lipophilic molecules by introducing non-linear cornea permeability–lipophilicity dependencies. Different calculated lipophilicity parameters and their squared values were investigated (***MLOGP***, ***WLOGP***, ***iLOGP***, log ***P***_ACD_, log ***P***_3.0_, ***Silicos-IT*** ***Log P***, ***Consensus*** ***Log P*** and ***ALogPS_logP***). It was determined that the best models (based on independent variables selected by forward stepwise regression, characterized by satisfying statistical parameters and, on the other hand, returning plausible log ***K***_corn_ values for studied pesticides) involve log ***D***_ACD_ (at pH = 7.4), ***XLOGP3*** and a quadratic term (***XLOGP3***)^2^. However, combined log ***D***_ACD_, ***XLOGP3*** and (***XLOGP3***)^2^ account for only ca. 57% of total log ***K***_corn_ variability, so additional independent variables were incorporated to produce Equations (14) and (15) presented below, which also include (i) ***PSA*** and electrotopological indices ***Se1C3O1d*** and ***SsNH2*** (Equation (14), Figure 3) and (ii) ***PSA***, calculated ***caco-2*** and ***MDCK*** permeabilities (Equation (15), Figure 4).

log ***K***_corn_ = −4.514 (±0.160) + 0.202 (±0.044) log ***D***_ACD_ + 0.477 (±0.081) ***XLOGP3*** − 0.0163 (±0.0024) ***PSA*** − 0.103 (±0.023) (***XLOGP3***)^2^ + 0.0627 (±0.0270) ***SsNH2*** + 0.0853 (±0.0253) ***Se1C3O1d*** (n = 60, R^2^ = 0.836, R^2^_adj._ = 0.818, F = 45.16, *p* < 0.01, RMSECV = 0.366, RMSEP = 0.415, Q^2^ = 0.792) (14)

log ***K***_corn_ = −6.365 (±0.954) + 0.244 (±0.040) log ***D***_ACD_ + 0.382 (±0.084) ***XLOGP3*** − 0.0911 (±0.0235) (***XLOGP3***)^2^
− 0.00985 (0.00197) ***PSA*** + 0.347 (±0.167) ***caco-2*** − 0.724 (±0.241) ***MDCK*** (n = 60, R^2^ = 0.830, R^2^_adj._ = 0.811, F = 43.11, *p* < 0.01, RMSECV = 0.329, RMSEP = 0.423, Q^2^ = 0.779) (15)

Equations (14) and (15) account for the reduced log ***K***_corn_ of more lipophilic compounds and the effect of ionization at physiological pH; the negative influence of ***PSA*** on cornea permeability is similar to that encountered for transport across other biological barriers, e.g., the blood–brain barrier [59,60] or skin [61]. Caco-2 permeability is measured on human colon epithelial cancer cells and the permeability through a monolayer of caco-2 cells is a common predictor of human intestinal absorption [62,63]; MDCK (Madin−Darby canine kidney) cells are also used to predict compounds’ membrane permeability [64]. Both cell lines are also used to study other absorption phenomena, e.g., blood–brain barrier permeability [65]. MDCK and caco-2 cell lines share many common epithelial cell characteristics; for passively transported compounds, MDCK and caco-2 experimental permeabilities are highly correlated. However, due to overexpression of the P-gp system in the MDCK cells, the apparent permeability coefficients (log ***P***_app_) in both cell lines differ for compounds susceptible to P-gp-mediated active transport [66].

**Figure 5:**
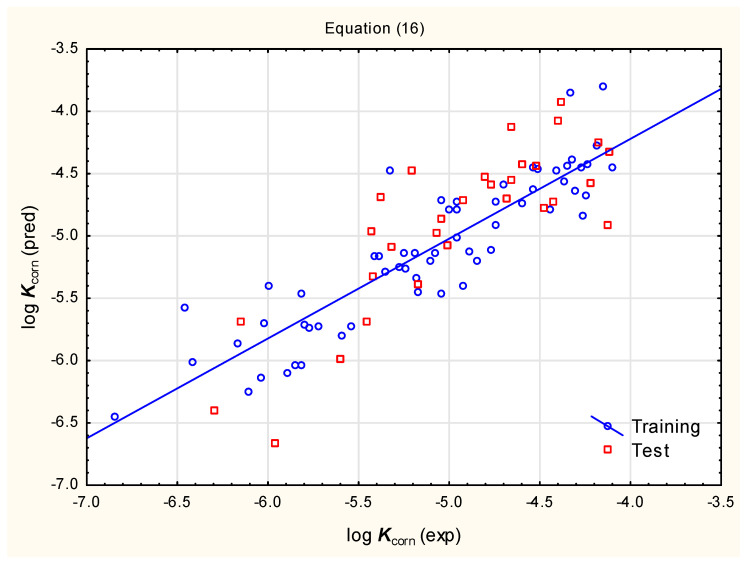
Predicted vs. observed log ***K***_corn_ for compounds **1** to **90**—Equation (16).

The calculated MDCK and caco-2 permeabilities (descriptors ***MDCK*** and ***Caco-2***) for compounds investigated in this study (training and test sets, ***1*** to ***69***; 348 pesticides from different chemical families) are not correlated (R^2^ = 0.189), so they could be incorporated into a single equation. In fact, considering the opposite signs of coefficients for both variables in Equation (15), it may be assumed that it is the difference between the caco-2 and MDCK permeabilities that is responsible for the slight improvement in the model’s fit and accounts for ca. 3% of the total log ***K***_corn_ variability, compared to the simplified Equation (16) (Figure 5).

log ***K***_corn_ = −4.542 (±0.141) + 0.220 (±0.042) log ***D***_ACD_ + 0.375 (±0.079) ***XLOGP3***
− 0.0860 (±0.0231) (***XLOGP3***)^2^ − 0.0120 (0.0015) ***TPSA*** (n = 60, R^2^ = 0.801, R^2^_adj._ = 0.785, F = 54.87, *p* < 0.01, RMSECV = 0.336, RMSEP = 0.368, Q^2^ = 0.762) (16)

Equations (14)–(16) give fairly similar results of predictions for the studied pesticides, but Equation (14) has the best statistical parameters, and for this reason, it was selected for further consideration. The compounds in the training and test sets are within the lipophilicity range ***XLOGP3*** ca. −1 to 4. Based on similarities between different biological barriers, it was assumed that the applicability domain for Equation (14) covers the ***XLOGP3*** range ca. −1 and 8; the log ***K***_corn_ values across this lipophilicity range are between ca. −6 and −4. In the studied group of pesticides, there are only two compounds outside of these limits; their predicted log ***K***_corn_ values are very low (−10.01 for glyphosate and −11.82 for paraquat, respectively). Such predicted values of log ***K***_corn_ for glyphosate and paraquat imply that their cornea permeability is very low. In reality, it can be suspected that (similarly to, e.g., ***BCF***) very highly hydrophilic or lipophilic compounds reach constant, low values of log ***K***_corn_, but there is insufficient experimental data to determine these values and all predictions in this respect would be highly speculative.

### 3.2. Rules of Thumb for Ophthalmic Drugs (RO_x_)

As stated above, based on the analysis of 145 marketed ophthalmic drugs, it was determined that the optimum physico-chemical parameters known to facilitate drugs’ efficient topical ophthalmic delivery are determined by a set of simple rules going by the name of “RO_x_” [14]:

Rule #1: distribution coefficient at physiological pH clog ***D***_pH7.4_ < 4.0.
Rule #2: topological polar surface area ***TPSA*** < 250 Å^2^.
Rule #3: free energy of octanol–water distribution Δ***G***_o/w_ < 20 kJ/mol.
Rule #4: sufficient aqueous solubility ***S*** > 1 mM.

It was reported earlier that the majority of pesticides adhere to Lipinski’s Ro5 [14,67,68] and those pesticides that meet the requirements of the Ro5 are far more likely to cross the placenta than those that do not [69]. With this in mind, we turned our attention to rules proposed for ophthalmic delivery (RO_x_); compounds **1** to **90** and 348 studied pesticides were analyzed in the context of these guidelines (as reported in the Supplementary Materials) and the following was determined:

- Compounds **1** to **90** fulfill Rules #1 to #3 (with one exception—cinoxacin, **53**).
- Pesticides fulfill Rule #2 without exception.
- There are three highly hydrophilic pesticides (from the “miscellaneous” group: glyphosate, paraquat and schradan) that do not fulfill Rule #3.
- There are several pesticides that breach Rule #1, but the percentage of such cases varies among the chemical families (carbamates—15%; organochlorines—83%; organophosphorus compounds—29%; pyrethroids—93%; triazines—3%; miscellaneous—40%).
- A large proportion of compounds (both pesticides and drugs from the training/test sets) violate Rule #4. This violation, however, seems to be a minor issue compared to violations of Rules #1 to #3; according to Karami et al. [14], the solubility range for ophthalmic drugs is relatively broad, with the lower limit of ca. 1 µM.
- There are pesticides whose calculated aqueous solubility is below 1 µM. Such a low solubility is usually associated with high lipophilicity. Such compounds, which in fact violate two RO_x_ rules, are expected to cross the cornea with difficulty, but their predicted log ***K***_corn_ is sufficient to facilitate some trans-corneal transport.

RO_x_ rules are crude filters generated on the basis of properties of existing ophthalmic drugs, and similarly to Lipinski’s Ro5 [67,68], they seem to give many false-negative results, especially if they are taken too strictly. However, Karami et al. [14] suggested that (considering how many deviations from their rules they observed) the violations of RO_x_ should be treated as “warnings”; the ideal drug candidates for ophthalmic delivery should adhere to the RO_x_ guidelines but compounds that do not may still exhibit some degree of corneal permeability, sufficient to be active inside the eyeball.

### 3.3. Pesticides’ Eye-Corrosive Potential

Although levels of the predicted cornea permeability for many pesticides appear to be very low, we knew that some of these compounds, although not entering the eye very easily, may still be dangerous for human and/or animal vision. Toxic effects of pesticides on the eyes in humans are mainly external, resulting from direct ocular exposure, but cases of ocular damage caused by systemic exposure have also been reported [44]. Qualitative (high/low irritation or corrosion potential) or quantitative (scoring from the Draize Eye Irritation Test [70]) indices of eye damage by chemicals are measured in vivo, ex vivo [71] or in vitro [72,73]. In silico predictions of chemicals’ eye irritation or corrosion potential involve principal component analysis or different machine learning algorithms, e.g., support vector machines or Bayesian neural networks [45,71,74,75,76,77,78,79,80,81,82,83]. In vitro and in silico models are important in the field of eye toxicity testing, as they are likely to substitute in vivo animal studies to some degree, thus contributing to the elimination of methods that are undesirable in the context of animal welfare.

In this study, we evaluated the corrosive potential of the studied pesticides using novel artificial neural network (ANN) binary (Yes—1; No—0) models of eye corrosion based on a large dataset collected by Wang et al. (2299 compounds) [45]. The ANN models we propose are based on a set of straightforward, self-explaining descriptors (listed in order of decreasing importance, based on Global Sensitivity Analysis scores > 1): ***MW***, ***PSA***, ***NH***, ***O***, ***N***, ***DONORS***, ***ALogPS_logP***, ***ALogPS_logS***, ***ACCEPTORS***, ***HALOG***, ***P*** and ***S***. The ANNs of different architectures (presented in Supplementary Materials) obtained at this stage of the study predict ca. 96% of cases correctly (in the training, test and validation groups), with almost equal percentages of false-negative and false-positive predictions (ca. 4%) (Table 1). Applied to the new compounds (348 pesticides), the networks return the expected “1” (or “Yes”) result, e.g., for glyphosate and paraquat, whose cornea-damaging activity is known [44].

## 4. Conclusions

A total of 348 pesticides were investigated in the context of their ability to cross the cornea, using novel in silico models based on a set of compounds whose experimental permeability coefficients are known. It was established that the physico-chemical properties of a large number of studied pesticides are outside the ranges typical of drugs used to train and test models. Extrapolation of linear models to predict the cornea permeability of highly lipophilic compounds (e.g., pesticides from the families of pyrethroids and organochlorines) leads to suspiciously high permeability values, implying very easy transport across the cornea. It was assumed, by analogy to other absorption/transport phenomena (e.g., bioconcentration in aquatic organisms), that the relationship between cornea permeability and lipophilicity is likely to be non-linear. The core of our improved models, accounting for the decrease in cornea permeability of highly lipophilic pesticides (log ***P*** up to ca. 8), involves (i) a quadratic function of lipophilicity (***XLOGP3***)^2^, (ii) the octanol–water distribution coefficient log ***D*** at pH 7.4 to account for compounds’ ionization in physiological conditions; and (iii) polar surface area (this parameter, according to Karami, should not exceed a certain limit for compounds absorbed across the cornea, e.g., ophthalmic drugs [14]).

Regarding the initial question—“How serious is the problem of pesticides’ cornea permeability?”—we have concluded (based on our predictions and the rules of thumb proposed by Karami [14]) that the majority of studied pesticides are likely to cross the cornea, but this process seems to be limited for many compounds, especially from the chemical families of organochlorines or pyrethroids. In the case of almost all the pesticides, however, if they come into direct contact with the eye surface as liquids, aerosols or vapors, there is a risk of ocular surface damage which can trigger a range of symptoms such as itching, hyperemia, photophobia or blurred vision. In more severe cases (especially after prolonged contact), the effects may include the development of dry eye disease, corneal ulcers, cataracts, glaucoma and impaired vision [43]. Theoretical predictions of eye corrosion potential (EC) of the studied pesticides (Supplementary Materials) based on artificial neural network models (built upon a large group of diverse compounds whose experimental potential to cause eye corrosion is known) show that eye-corrosive cases are especially abundant among the pesticides from organochlorine and organophosphorus families. The requirements regarding labeling and safety precautions for pesticides vary according to the region. Many such compounds are (or should be) labeled as “causing serious eye damage”, “causing eye irritation” or “causing serious eye irritation” (H318, H320 or H319 health phrases according to [84]). In many countries (e.g., in Poland), they can only be sold and used by authorized persons who have completed special courses and can provide the participants with proper understanding of pesticides’ safety, application techniques and regulatory compliance.

Some pesticides (e.g., paraquat or glyphosate) are known to be toxic to the ocular surface [44]; for others, their impact on ocular health is just being discovered. Extreme caution and the use of eye protection must be recommended to avoid contact with the ocular surface, and further studies on the effects of such contacts are underway.

## Figures and Tables

**Table 1:** Classification summary (MLP 12-13-2; training algorithm BFGS 50; error function—entropy; hidden activation—Tanh; output activation—Softmax).

	Training		Test		Validation		Total		
	Class-0	Class-1	Class-0	Class-1	Class-0	Class-1	Class-0	Class-1	Class-All
Total	994	617	210	134	208	136	1412	887	2299
Correct	964	592	200	129	200	132	1364	853	2217
Incorrect	30	25	10	5	8	4	48	34	82
Correct (%)	97.0	95.9	95.2	96.3	96.2	97.1	96.6	96.2	96.4
Incorrect (%)	3.0	4.1	4.8	3.7	3.8	2.9	3.4	3.8	3.6

## Data Availability

Data reported in the manuscript.

## Supplementary Materials

The following supporting information can be downloaded at https://www.mdpi.com/article/10.3390/pharmaceutics17020156/s1.

## Abbreviations

***SsNH2***	atom type e-state descriptors, number of ssNH2
***SaaCH***	atom type e-state descriptors, sum of aaCH
***Se1C1C3a***	single bond-type e-state index
***Se1C3O1d***	single bond-type e-state index
***NH***, ***O***, ***N***, ***DONORS***, ***ACCEPTORS***, ***HALOG***, ***P*** and ***S***	counts of particular atoms
***ALogPS_logS***	aqueous solubility
log ***D***_ACD_	octanol–water distribution coefficients at pH = 7.4
***ALogPS_logP***; log ***P***_ACD_; ***XLOGP3***; ***iLOGP***; ***MLOGP***; ***WLOGP***; ***Silicos-IT Log P***; ***Consensus Log P***	octanol–water partition coefficients calculated according to different algorithms
***MW***	molecular weight
***Vol***	van der Waals volume
***nHD***	H-bond donor count (OH + NH)
***nHA***	H-bond acceptor count (N + O)
***fChar***	formal charge
***nRig***	rigid (non-flexible) bond count
***nRot***	rotatable bond count
***Flex***	flexibility = ***nRot/nRig***
***nStereo***	stereocenter count
***MaxRing***	maximum ring size
***nHet***	number of heteroatoms, i.e., non-carbon atoms including hydrogens
***F_Csp3_***	fraction of sp^3^ carbons
***PSA***	polar surface area
***TPSA***	topological surface area
***PAMPA***	parallel artificial membrane permeability assay
***MDCK***	Madin−Darby canine kidney cells’ logarithmic permeability coefficient
***Caco-2***	human colon adenocarcinoma cell lines’ permeability
RMSEP	root mean square error of prediction (external test set)
RMSECV	root mean square error of cross-validation
Q^2^	leave-one-out cross-validated R^2^
